# Relative Position of Chloroform, Etc., in Obstetric Practice

**Published:** 1876-04

**Authors:** Philip Adolphus

**Affiliations:** Linical Instructor in Gynecology, Rush Medical College; Attending Physician for Diseases of Women and Obsterics, Central Dispensary, Chicago


### THE RELATIVE POSITION OF CHLOROFORM, SULPHURIC ETHER, AND SULPHATE OF MORPHIA IN OBSTETRIC PRACTICE.

By PHILIP ADOLPHUS, M.D.,

LINICAL INSTRUCTOR IN GYNECOLOGY, RUSH MEDICAL COLLEGE; ATTENDING PHYSICIAN FOR DISEASES OF WOMEN AND OBSTERICS, CENTRAL DISPENSARY, CHICAGO.

(Read before the Chicago Medical Society, January 17, 1876.)

The introduction in 1847, by the illustrious Simpson, of Sulphuric Ether and Chloroform, as anæsthetics in midwifery, forms an era in the practice of medicine.

He established rules for their inhalation, and defended their administration, although attacked by many eminent men.

Nearly thirty years have elapsed, and the principles and facts he then stated, have in general been endorsed by the medical world.

The experience of others has modified the use of anæsthetics in labor in some respects. However, this illustrious man will always receive grateful recognition ; for, his application of agents, to uses before unknown, has saved women untold pangs of agony. Moreover, his persevering and triumphant defense of the maxim, that *pain is a non-essential element of parturition*, and his splendid practical demonstration of the same, has still further increased our obligations to him.

The experience of the profession in the use of anæsthetics in midwifery, agrees with the conclusions of Simpson, in the following points:

It diminishes the perils, and removes the pains of labor ; it husbands the strength of the patient, and wards off nervous depression ; relaxes the soft parts, thus doing away with the resistance offered to the expulsion of the head, by the os uteri, vagina, and the muscles of the perineum. It renders recoveries after labor more rapid and perfect; while its effects do not seem to be injurious to the child.

Anæsthesia is strongly urged in cases of difficult instrumental labor, in turning, when the waters have become drained, in cases of convulsions, rupture of the uterus, etc.

Under the influence of anæsthetics, these operations are performed with greater ease and facility to the physician, and consequently with greater safety to the patient. In such cases it will always be necessary to push anæsthesia to the surgical degree.

The use of sulphuric ether, however, is at present preferred by the profession to that of chloroform; not merely in ordinary labors, but in those cases of instrumental deliveries, in which anæthesia is pre-eminently indicated. It is safer, and is free from the objections to which the use of chloroform in surgery is liable. With Elliot, it may be stated that "a man having the misfortune to lose a patient by an anæsthetic, would have more sympathy and approval if he had used sulphuric ether, than if he had selected chloroform."

Even the impregnable position which chloroform has so long maintained in midwifery, must give way before the actual experience of danger, during its inhalation. For Dr. Depaul says that, "accidents from obstetrical administration of chloroform are not unknown. He is in possession of cases in which sudden death has been produced by it. He believes it requires great care in its administration, and in ordinary labors can be dispensed with." "In the New York Obstetrical Society, 1874, Dr. Lusk reported two cases, which came near being fatal from the use of chloroform during labor." (Quarterly Report A. I. Obsterics, May, 1875.)

Elliot found the pulse to intermit, and become depressed under chloroform in one case.; and in another (case 22, obstetric clinic 1868), his patient experienced alarming symptoms from the use of chloroform in a natural labor.

Although it is unquestionably true, that the pains of labor induce toleration of an anæsthetic, it is not now considered expedient to carry it in ordinary labors to complete anæsthesia; for, moderately administered, it will quiet nervous irritation, restlessness, irregular spasms, and delirium. Furthermore, to avoid inefficiency of uterine contractions which frequently occurs when complete anæsthesia is established, it is sufficient to produce a slight anæsthetic influence, to moderate, without destroying, sensibility; so that labor progresses regularly. It is expedient also to preserve consciousness, so that command of the voluntary powers of the patient may be maintained during the progress of labor.

There are, however, objections to the employment of both ether and chloroform in natural labor, which have received just consideration at the hands of eminent observers. This has induced them to *restrict their administration to cases of instrumental deliveries, and those in which there is some serious complication.* For, ordinarily, these agents may accomplish too much, by diminishing the force and frequency of uterine contractions; and sometimes predisposing to post-partum hæmorrhage. They blot out memory, cut off perception, and lessen reflex irritability, when more or less consciousness is wanted. The results of their administration are feared by most patients, for the many deaths from chloroform in surgical practice, and its consequent rejection by the profession, have become known. Moreover, their administration awakens the solicitude of attendants, and requires the constant attention of the physician to the state of the pulse, breathing and heart. Finally, the stage of excitement, especially that produced by ether, is annoying.

To conduct a given case of labor to a happy termination, we need an agent that does not diminish the strength or intensity of the uterine contractions, but lessens their painfulness, and one which is also applicable to all periods of labor. We require of it the relaxation of tissues, lessening and overcoming of spasms, production of drowsiness—not quite sleep, but nearly so—that the approaching pain may rouse the patient, who suffers comparatively little.

*We possess this agent in sulphate of morphia,* (which I have used in these cases, during the past eighteen years.) It diminishes reflex irritability, produces delightful calmness, induces sleep, and does not obliterate consciousness or intelligence. It calms restlessness and spasms, relieves pain, irregular muscular contractions and convulsive movements; it removes congestions by diuresis and diaphoresis; and, above all, labor is not interrupted during its action; nay, uterine contractions are sometimes increased by it, owing to the removal of all irritation.

"It acts primarily on the peripheral ends of the sensory nerves; partly, also, on the sensitive fibres in their course; and probably, also, on the central brain elements. The muscles and the motor nerves suffer no change of function from its action." (Waldemar Baxt. St. Petersburg, Arch. f. Anat. u. Physiol., No. 2, 1869; Journ. Am. Sciences, Oct., 1869.)

Its administration, when supplemented by frequent sponging of face and hands with cold water and eau de cologne, use of the fan, administration of pellets of ice, and encouragement by comforting assurances, brings ordinary labors, of whatever duration, to a happy termination, without exhaustion of the system, and without the occurrence and consequences of the nervous shock attendant upon delivery, which so much enhance the danger and fatality of childbed.

Nay more; should manual or instrumental interference become necessary—provided a skillful physician has previously superintended the accouchement—we find our patient in good condition, mental as well as physical, ready and willing to undergo the operation. We have the advantage, if ether can be dispensed with—and in many forceps deliveries and cases of turning it may be omitted—of securing the co-operation of our patient, which is so valuable an auxillary.

It is, moreover, a fact worth remembering, that when the administration of ether or chloroform becomes necessary, a comparatively small quantity will induce and maintain anæsthesia ; provided, a preliminary administration of a full dose of morphia has preceded it. Anæsthesia is, under those conditions, induced without any initiatory stage of excitement, and is very complete.

It is not necessary to precede the administration of morphia, by venesection, as is insisted by many authorities. It is true that the circulation is more active, and even sometimes becomes exaggerated both in pregnancy and parturition, giving rise in some women to vertigo, dimness of vision, ringing of the ears, sudden flushing of the face, and spontaneous heat over the body and head. But these symptoms, formerly attributed to plethora, are, in reality, evidences of poverty of the blood; for Andral and Gavarret have shown that its corpuscles are sensibly diminished, and the fibrin and watery portion increased. All of this is characteristic of anæmia and chlorosis; whilst the increase of blood corpuscles is the essential characteristic of plethora.

These alterations of the blood in pregnancy, are physiological, but may so increase as to become pathological. We have, consequently, in pregnant women, the different neuralgias, and the same modifications of circulation, which we find in chlorotic and anæmic women. Venesection is, therefore, not to be practiced in ordinary cases of labor. The bearing-down efforts during parturition, undoubtedly disturb respiration and circulation : deep, full inspirations will, however, relieve uneasy sensations, congestion of the lungs, heart, and even of the brain. Tartar emetic, in minute doses, added to the morphia, exerts a happy influence in removing congestion, when present.

My mode of administering morphia during labor, is as follows: A teaspoonful of a solution containing one-sixth of a grain (or five drops of Magendie's solution of morphia) is given whenever the patient complains of unusual pain or irritation, *and then only.* This is repeated, as often as is necessary to insure calmness, comfort and the due progress of labor. The agonizing pains of the last stage are endured with equanimity and fortitude; the patient often sleeping during the short intermissions of pain, provided it has been administered with skill and firmness. From four to ten doses administered at intervals during the course of the labor, generally prove sufficient. Previous to delivery, a teaspoonful of fluid-extract of ergot, with the addition of one-sixth of a grain of morphia, is given; and the patient, after its completion, feels comfortable, is not exhausted, and obtains a few hours sleep.

This mode of administering morphia, will obviate the exhaustion, which is produced by emotion, fear, and shrinking from pain, and which frequently delays labor. Furthermore, *the contractions are sometimes increased in force and regularity, without being rendered more painful.*

Thus administered, it obviates the rigidity of the soft tissues, which is frequently found in women of nervous temperaments, resulting, when not prevented, in tedious labors, which are dangerous to mother and child.

The results of this rigidity are, in the case of the mother, exhaustion of power, augmented pains of uterus, severe cramps, nervous delirium and fever; tumidity of the lips of the os uteri, sometimes followed by inflammation and its sequelæ, laceration of the cervix, uterus or perineum.

In the case of the child: where the liquor amnii has been evacuated, and the os uteri remains undilated, disturbances of the placental functions, and like disturbances in the circulation of the cord, and in the body of the child, take place, producing congestion, asphyxia, and death.

